# Tosylation of alcohols: an effective strategy for the functional group transformation of organic derivatives of polyoxometalates

**DOI:** 10.1038/s41598-017-12633-8

**Published:** 2017-10-02

**Authors:** Hongli Jia, Qi Li, Aruuhan Bayaguud, Shan She, Yichao Huang, Kun Chen, Yongge Wei

**Affiliations:** 1 Key Lab of Organic Optoelectronics & Molecular Engineering of Ministry of Education, Department of Chemistry, Tsinghua University, Beijing, 100084 P.R. China; 2 State Key Laboratory of Natural and Biomimetic Drugs, Peking University, Beijing, 100191 P.R. China

## Abstract

Recently, the organic functionalization of polyoxometalates (POMs) has drawn increasing interest, and an easy and effective route to achieve organic derivatives is of great importance. Herein, the first reported synthesis of a tosyl ester derivative of the polyoxometalate (Bu_4_N)_2_[V_6_O_13_{(OCH_2_)_3_CCH_2_SO_3_C_7_H_4_}_2_]·2.5CH_3_CN (compound **1**) was performed by using DMAP as an activating reagent and triethylamine as an HCl scavenger. The tosyl ester was transformed into an azide or halide group by using sodium azide or sodium bromide, respectively, as the nucleophilic agent. Two derivatives of POMs, (Bu_4_N)_2_[V_6_O_13_{(OCH_2_)_3_CCH_2_N_3_}_2_]·4CH_3_CN (compound **2**) and (Bu_4_N)_2_[V_6_O_13_{(OCH_2_)_3_CCH_2_Br}_2_] (compound **3**), were easily obtained. All the compounds were structurally and compositionally characterized by single-crystal X-ray diffraction, elemental analysis, IR spectroscopy, NMR spectroscopy, ESI-MS, UV-Vis spectroscopy and TGA. This work provides a new route for the functional group transformation of organic derivatives of polyoxometalates.

## Introduction

Tosylation of hydroxyl-functionalized substrates is an important transformation to activate hydroxyl groups, thus yielding substrates for further nucleophilic substitution in various fields of organic syntheses^1,2^. In general, the treatment of alcohols with tosyl chloride, an amine and a proper catalyst in an organic solvent is a conventional method to prepare tosylates^3^. The resulting sulfonic esters can subsequently be transformed to many other groups with an appropriate nucleophile^4,5^, such as alkali azides, thiocyanates, sulfonates^4^ and amines^5^. With various functional group interconversion, many natural products or drugs can be synthesized successfully^6,7^. Among them, organic azides, an important class of energy-rich and flexible intermediates^8^, have drawn considerable interest since the late 19th century^9^. Organic azides can act not only as precursors for the synthesis of amines^10^ and heterocycles, such as triazoles and tetrazoles^11,12^ but also as functional groups in pharmaceuticals, as exemplified by azido-nucleosides in the treatment of AIDS^13^. Additionally, organic azides can be bioconjugated via Staudinger ligation^14^ or click reaction^15^.

Polyoxometalates (POMs) are well-defined early transition metal-oxygen anionic clusters that exhibit unique chemical and physical properties^16^ and have been applied in many fields, including catalysis, optics, medicinal chemistry and materials science^17–19^. Owing to the multi-modifiable sites on POM surfaces, the covalent organic modification of POMs has made great progress in the past few decades, and large numbers of POM-based hybrid materials have been obtained^20^. Polyoxovanadates (POVs) are a very fast growing subclass of POMs, owing to their attractive catalytic oxidative^20^ and electronic properties^21,22^ as well as their biological activities^23,24^. To the best of our knowledge, the post-functionalization of pre-formed hybrid POV platforms with a reactive group, such as –OH or –NH_2_, is expected to be an increasingly attractive method to synthesize POV-based hybrid materials, because the direct functionalization of POVs usually produces particularly low yields of the hybrid products because of the strong oxidative properties of the high-valence vanadium precursors^25^. Among the well-known organic-inorganic hybrid POVs, pentaerythritol-derivatized hexavanadate (Bu_4_N)_2_[V_6_O_13_{(OCH_2_)_3_CCH_2_OH}_2_] has high importance in fabrication of novel POV-based hybrid materials. Pentaerythritol-derivatized hexavanadate is easily accessed, as demonstrated by the pioneering work by Zubieta *et al*.^26^. The recent development by Wei *et al*. has made large-scale synthesis possible^27^. With its two reactive hydroxyl groups, pentaerythritol-derivatized hexavanadate usually acts as a building block for further modification through simple esterification reactions^28–31^, and its targeted applications include catalysis^29^ and self-assembly of supramolecular structures^30^.

However, there has been only one report on tosylation involving the simple use of a POM as a catalyst^32^, and the tosylation of hydroxyl-functionalized organic derivatives of POMs has never been reported. Therefore, we sought to directly activate the hydroxyl groups of an organically derivatized POM through tosylation, to study the potential functional group transformations in POM chemistry. Because azide is an important and versatile functional group, many chemists have focused on grafting this ligand onto POMs to explore the further functionality of these two parts. For example, azide-functionalized Keggin- and Dawson-type^33–38^ polyoxotungstates, Anderson-type polyoxomolybdates^39,40^ and even Lindqvist-type^41^ polyoxomolybdates have been well studied, and they can serve as building blocks for further post-functionalization through click reaction^33–41^. However, azide-functionalized hexavanadate has been much less studied than polyoxomolybdates and polyoxotungstates. In this paper, beginning with the easily afforded pentaerythritol-derivatized hexavanadate containing two pendant hydroxyl groups as a starting material, p-toluene sulfonyl chloride as the sulfonyl source, DMAP as an activating reagent and triethylamine as the HCl scavenger, a tosyl ester derivative of a polyoxometalate, (Bu_4_N)_2_[V_6_O_13_{(OCH_2_)_3_CCH_2_SO_3_C_7_H_4_}_2_]·2.5CH_3_CN (compound **1**), was synthesized with a good yield (as high as 60%). Then, with the nucleophilic agent NaN_3_, the tosyl ester was transformed into an azide, and the corresponding (Bu_4_N)_2_[V_6_O_13_{(OCH_2_)_3_CCH_2_N_3_}_2_]·4CH_3_CN (compound **2**) was obtained. To demonstrate the universality of this protocol, a bromide group, a well-known organic group and a widely used key intermediate in various organic transformations^42,43^ were also applied to this protocol. Finally, a bromide-functionalized hexavanadate, (Bu_4_N)_2_[V_6_O_13_{(OCH_2_)_3_CCH_2_Br}_2_] (compound **3**), was also synthesized, thus confirming the feasibility of our synthetic strategy in organic functionalizations of POVs. These compounds were characterized by single-crystal X-ray diffraction analysis. Such tosyl ester derivatives of POMs might provide a potential route for the transformation of functional groups of organic derivatives of POMs.

## Results and Discussion

### Synthesis analysis

#### Functional group transformation in POMs

The steric hindrance and electron-withdrawing properties of POMs play negative roles in their synthesis. Thus, excess organic substrates, longer reaction times and higher temperatures are required, but low yields are obtained in the stepwise functionalization of POMs compared with those of common functional group transformation reactions in organic chemistry, which are usually performed at room temperature within several hours^2,3^. For the synthesis of extended functional-group-derivatized POVs, the following must be considered: (i) The yield is particularly low via the direct functionalization of POVs because of the strong oxidative properties of high-valence vanadium. Therefore, it is wise to adopt a stepwise functional group transformation procedure. (ii) Pentaerythritol-derivatized hexavanadate is easily accessed, and even large-scale synthesis is possible. Therefore, in this paper, tosylation of pentaerythritol-derivatized hexavanadate was used to prepare azide/bromide-functionalized hexavanadates.

**Figure 1:**
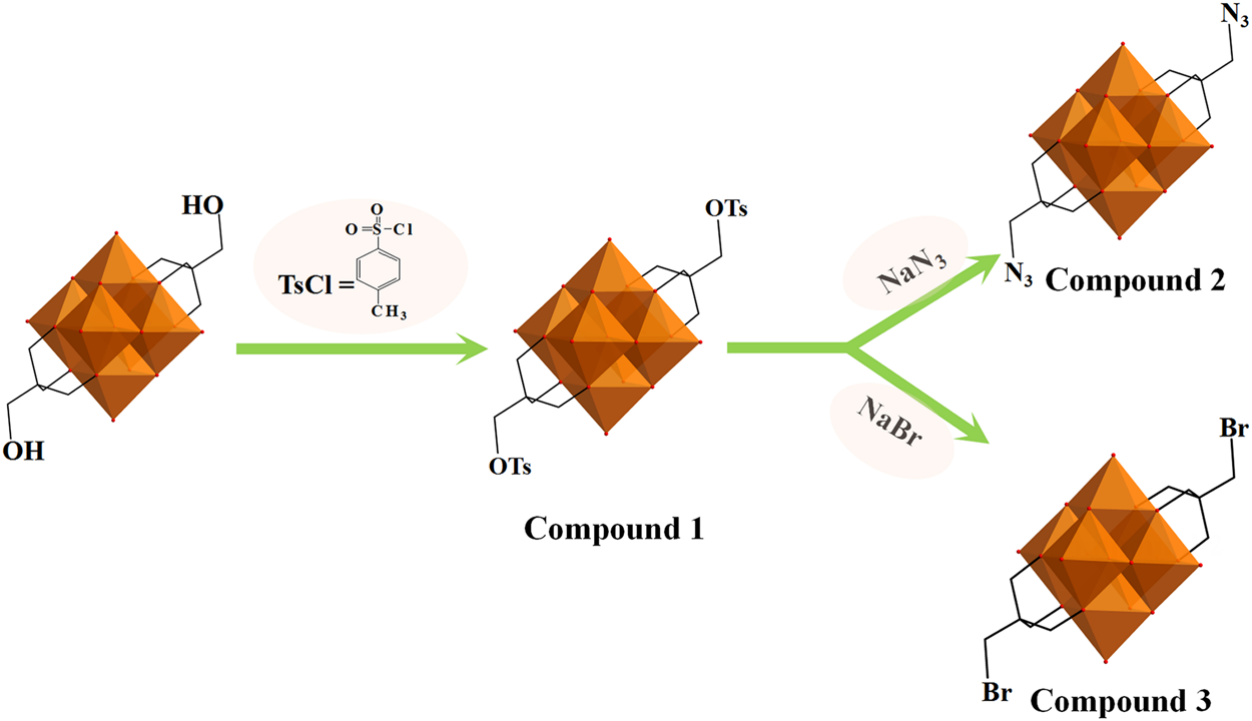
Step-by-step functional group transformation of POVs.

In Fig. 1, by using pentaerythritol-derivatized hexavanadate as the precursor, the hydroxyl group was activated first. The reaction of pentaerythritol-derivatized hexavanadate, tosyl chloride, DMAP and Et_3_N with a molar ratio of 1:2.2:2:2.85 in acetonitrile solution after heating at 50 °C for 2 days led to the formation of compound **1**. By simply filtering the mother liquor into water and collecting the precipitate, highly pure compound **1** was obtained. Here, Et_3_N acted as an HCl scavenger during the sulfonyl transfer reaction. Our attempt to use inorganic bases, such as K_2_CO_3_, produced disappointing results. Et_3_N has a substantial advantage over K_2_CO_3_ because it can disperse in acetonitrile homogeneously. DMAP, an activating reagent, was used to facilitate the reaction, owing to the similarities of sulfonyl and acyl transfer. Notably, compared with common organic tosylation with a low dose of DMAP, here in this work, 2 eq. of DMAP was needed. With a smaller amount of DMAP, the tosylation of POVs failed. In addition, sulfonyl chloride is moisture sensitive, so dry acetonitrile was used to prevent the hydrolysis of the chloride. Moreover, a mixture of single-sided and double-sided tosylated products will form if the sulfonyl chloride precursor is inadequate. However, these products can be separated conveniently by recrystallization, from which the double-sided tosylated products crystalize first. The yield was only approximately 60%, a value inferior to that for common tosylation in organic reactions. However, it was comparable to the yield of common post-functionalized hexavanadate hybrid products, which have yields in the range of 10–65%^28–31^. In the second step, N_3_^−^ and Br^−^ were chosen as the nucleophilic agents to study functional group transformations in POVs. Thus, compound **2** was obtained first by the reaction of pentaerythritol-derivatized hexavanadate with NaN_3_ in DMF solution in a molar ratio of 1:8 at 80 °C for 2 days. Fortunately, compound **2** was easily crystallized in the mother liquor with a yield of 68% by addition a small amount of acetonitrile and water. To confirm the feasibility of the functional group transformation strategy applied to POVs, Br^−^ was used as a nucleophile to attack the sulfonic acid ester groups of compound **1**. Even though a higher temperature, i.e., 90 °C, was used for compound **3**, the yield was still lower than that of compound **2**. This result was ascribed to the poor nucleophilicity of bromide. Although the yields of compounds **2** and **3** were less than 70%, they were higher than that of carboxyl-derivatized hexavanadate, which is a functional group transformation compound obtained via the oxidation of a pentaerythritol-derivatized hexavanadate precursor at a yield of 55%^44^. Notably, an 8-fold excess of NaN_3_ or NaBr was required to circumvent the single-sided products. Briefly, tosylation of alcohols followed by nucleophilic substitution is a well-established process in organic chemistry. However, owing to the strong electron-withdrawing properties of POMs, tosylated derivatives of POMs are less likely than pure organic substrates to undergo nucleophilic substitutions. Through this step-by-step procedure (Fig. 1), the desired compounds were obtained, and the yields were ca. 40% for the final POVs products. This work demonstrated that tosylation is a promising method for functional group transformations of POVs.

### Structure description

**Figure 2:**
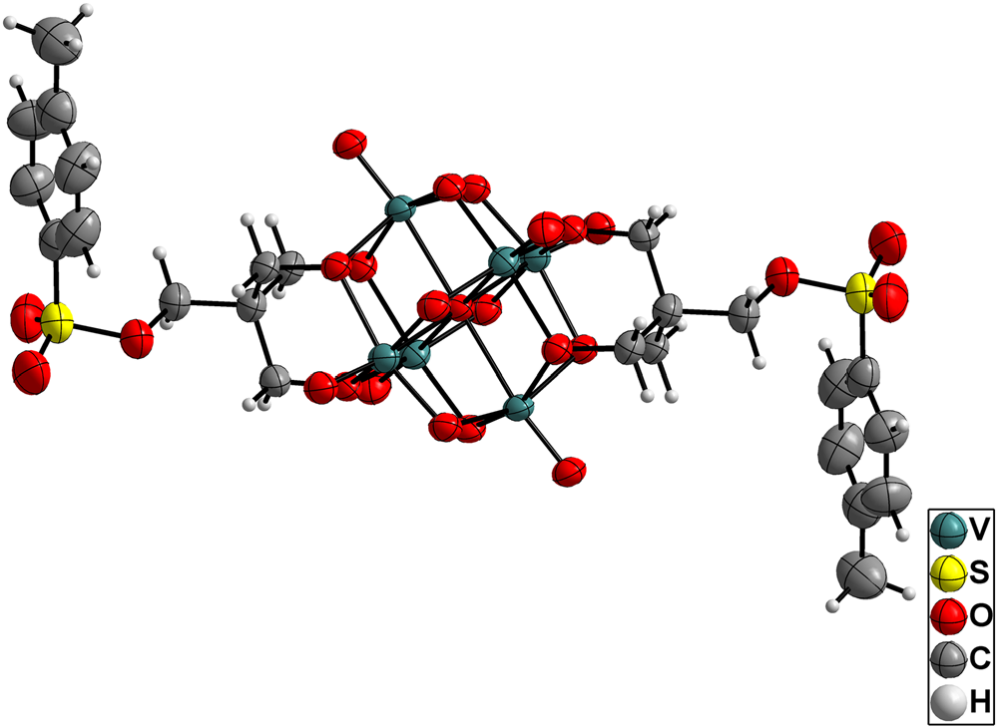
ORTEP drawing of the anionic cluster of compound **1**. Thermal ellipsoids are drawn at the 50% probability level. Colour scheme: V = teal; S = yellow; O = red; C = grey; H = light grey.

**Figure 3:**
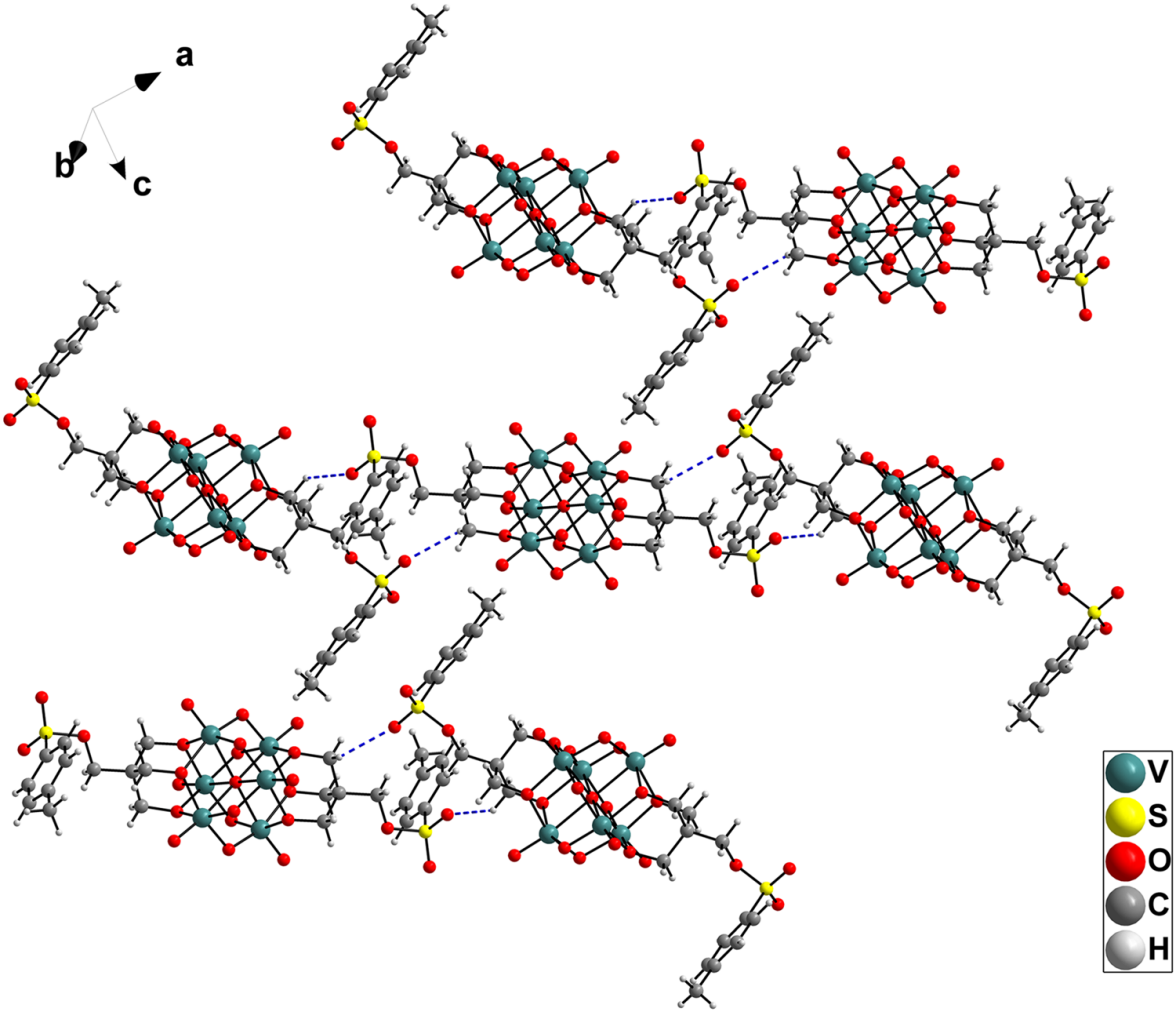
The 2D supramolecular layers (bottom) of compound **1**. Colour scheme: V = teal; S = yellow; O = red; C = grey; H = light grey.

**Figure 4:**
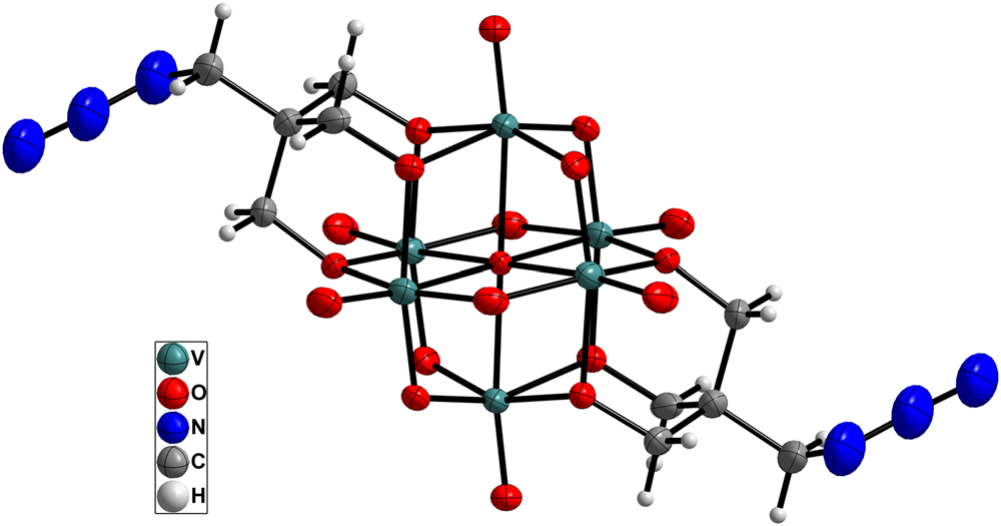
ORTEP drawing of the anionic cluster of compound **2**. Thermal ellipsoids are drawn at the 50% probability level. Colour scheme: V = teal; N = blue; O = red; C = grey; H = light grey.

**Figure 5:**
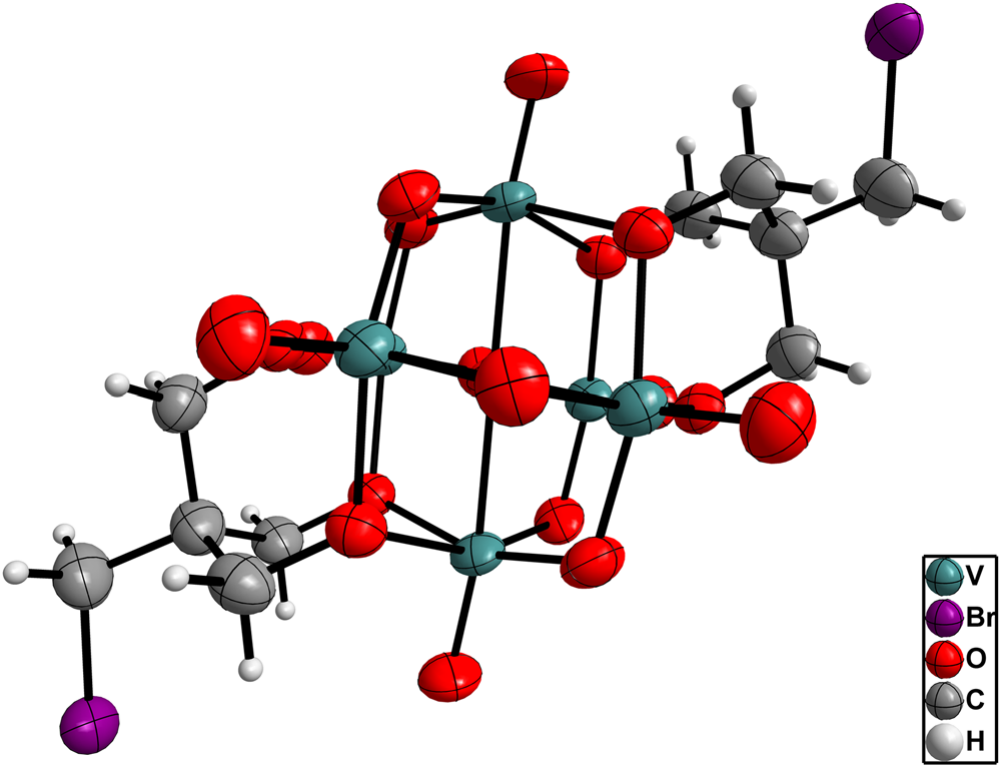
ORTEP drawing of the anionic cluster of compound **3**. Thermal ellipsoids are drawn at the 50% probability level. Colour scheme: V = teal; O = red; Br = purple; C = grey; H = light grey.

The structures of compounds **1**–**3** were validated by single-crystal X-ray diffraction. The crystallographic data for the compounds are summarized in Table 1. Compounds **1** and **2** crystallized in the monoclinic system, *C2/c* space group, whereas compound **3** crystallized in the triclinic system, *P*-1 space group. The anionic structures of these compounds were all derived from Lindqvist-type POVs, in which the six vanadium atoms form an octahedron, and two tris(alkoxo) ligands occupy the opposite faces of the octahedron. In compound **1**, the tosyl group is connected to the hexavanadate cluster through a sulfonic acid ester bond (1.583 Å) (see Fig. 2). Interestingly, the sulfonyl oxygen can participate in intermolecular hydrogen bonding to the carbon atom of the adjacent hexavanadate cluster (C10–H10B…O1 = 3.117 Å, see Table S5). Thus, the anionic cluster propagates into a supramolecular dimer. Moreover, the adjacent phenyl rings from the neighbouring dimers form face-to-face π…π packing with a distance of 3.72 Å (see Figure S1) and then extend to form a 2D layered structure (Fig. 3). The selected bond lengths, bond angles and torsion angle of compound **1** are listed in Tables S1 and S4. The anionic structure of compound **2** was derived from compound **1**, such that the sulfonic acid ester groups are replaced by two azide groups (see Fig. 4). Owing to the disorder of the organic moiety, only some of the bond lengths and bond angles are given here (see Table S2). For compound **3**, the bromide groups are attached to hexavanadate by nucleophilic attack of the sulfonic acid ester groups of compound **1** (see Fig. 5). However, because of the disordered bromide groups, some bond lengths and bond angles are not given here (see Table S3). Thus, the tosylation reaction is an effective strategy for the functional group transformation of organic derivatives of polyoxometalates.

**Table 1:** Crystallographic data and structure refinement for compounds **1**–**3**.

	1	2	3
Empirical formula	C_61_H_109.5_N_4.5_O_25_S_2_V_6_	C_50_H_100_N_12_O_19_V_6_	C_42_H_88_Br_2_N_2_O_19_V_6_
Size [mm^3^]	0.1 × 0.2 × 0.3	0.4 × 0.35 × 0.15	0.15 × 0.2 × 0.35
Formula weight	1675.79	1478.55	1390.60
Crystal system	Monoclinic	Monoclinic	Triclinic
Space group	*C2/c*	*C2/c*	*P*-*1*
*a* [Å]	29.7672(12)	28.0514(15)	10.8872(2)
*b* [Å]	16.4119(4)	16.1942(6)	13.0882(4)
*c* [Å]	16.6684(5)	16.8286	20.4111(6)
*α* [°]			81.547(3)
*β* [°]	108.645(3)	115.432(4)	83.464(2)
*γ* [°]			85.946(2)
*V* [Å3]	7715.7(4)	6903.9(5)	2853.86(14)
*Z*	4	4	2
Dcalc [g.cm^−3^]	1.443	1.379	1.618
Temperature [K]	173.0(1)	106.1(2)	297.51(18)
Tmax/Tmin	1.000/0.504	1.000/0.899	1.000/0.577
Absorption coeff. [mm^−1^]	6.992	0.849	10.131
*F* (000)	3508.0	3008	1428.0
*θ* range [°]	3.84 to 76.32	2.99 to 29.60	3.42 to 75.95
Reflections collected	27383	13862	20449
GOF on *F* ^*2*^	1.048	1.055	1.052
Final *R* indices [*I* > 2σ(*I*)]	*R*_1_ = 0.0509, *wR*_2_ = 0.1456	*R*_1_ = 0.0632, *wR*_2_ = 0.1625	R_1_ = 0.0685, *wR*_2_ = 0.2029
*R* indices (all data)	*R*_1_ = 0.0562, *wR*_2_ = 0.1510	*R*_1_ = 0.0862, *wR*_2_ = 0.1781	R_1_ = 0.0834, *wR*_2_ = 0.2153

^†^ *wR*_2_ = {Σ[w(*F*_o_^2^ − *F*_c_^2)2^]/Σ[w(*F*_o_^2)2^]}^1/2^; *R*_1_ = Σ| |F_o_| − |F_c_| |/Σ|Fo. *GooF = *S* = {Σ [*w*(*F*_o_^2^ − *F*_c_^2)2^]/(n − p)^2^}^1/2^.

#### Spectroscopic characterization

The IR spectra of compounds **1**–**3** were recorded in detail (Figures S2–S4). In the IR spectra, the typical stretches of the parent hexavanadate clusters of these compounds were very similar and were located in the range of 1000–700 cm^−1^ ^45^. The peaks at 1130 and 1065 cm^−1^ in compound **1**, 1121 and 1058 cm^−1^ in compound **2** and 1112, and 1058 cm^−1^ in compound **3** were assigned to the vibration peaks of the C–O bonds, thus demonstrating the grafting of the triol ligand onto the surface of the POVs^45^. For compound **1**, the characteristic peak at 1176 cm^−1^ corresponded to the –SO_3_ vibration^46,47^, whereas for compound **2**, the appearance of strong broad peaks at 2101 cm^−1^ arose from N_3_^−^ vibrations^11^, thus indicating that N_3_^−^ was introduced into the final frameworks successfully. All these features were consistent with the X-ray structure analysis. In addition, UV-Vis spectroscopy of compounds **1**–**3** showed that all the clusters feature O–V ligand-to-metal charge-transfer (LMCT) bands in the UV range.

#### NMR spectra

In the ^1^H NMR spectra of compounds **1**–**3**, all the signals were clearly resolved and unambiguously assigned. The proton signals of the two tetrabutylammonium cations (Bu_4_N^+^) were present at 0.9–3.10 (Figures S5, S7 and S9). In compound **1**, the peak at 4.87 ppm corresponded to –CH_2_O groups, whereas the singlet peak at 3.83 ppm represented the –CH_2_ group directly linked to the sulfonyl group from the benzene ring. The region from 7.41 to 7.75 ppm corresponded to the phenyl aromatic signals. For compound **2**, The observed sharp singlet peak at 4.84 ppm corresponded to –CH_2_O groups, which are directly covalently attached to the POM. In addition, –CH_2_N_3_ groups appeared at 3.39 ppm. The ^1^H NMR spectrum of compound **3** showed a pattern quite similar pattern to that of compound **2**, in which the –CH_2_O and –CH_2_Br groups were located at 5.03 and 3.35, respectively. In the ^13^C NMR spectra, all the signals were also clearly resolved and unambiguously assigned (Figures S6, S8 and S10). Intriguingly, owing to the heavy atom effect of Br^48^, the chemical shifts of the methylene and quaternary carbons shifted downfield relative to those in compound **2**. The results of the NMR analyses also supported the structures of the organic moieties of the derivatives.

#### ESI-MS spectra

The peaks in the ESI mass spectra were assigned to the corresponding ion pairs (Figures S11–S13). For compound **1**, the peaks observed at m/z 543.86 and 1330.00 corresponded to [V_6_O_13_{(OCH_2_)_3_CCH_2_SO_3_C_7_H_4_}_2_]_2_^−^ and {(Bu_4_N)[V_6_O_13_{(OCH_2_)_3_CCH_2_SO_3_C_7_H_4_}_2_]}^−^, respectively. Similarly, for compound **2**, the peaks at m/z 414.85 and 1071.99 were assigned to [V_6_O_13_{(OCH_2_)_3_CCH_2_N_3_}_2_]^2−^ and {(Bu_4_N)[V_6_O_13_{(OCH_2_)_3_CCH_2_N_3_}_2_]}^−^, respectively. For compound **3**, the peaks at m/z 452.76 and 1147.81 were ascribed to [V_6_O_13_{(OCH_2_)_3_CCH_2_Br}_2_]^2−^ and {(Bu_4_N)[V_6_O_13_{(OCH_2_)_3_CCH_2_Br}_2_]}^−^, respectively.

#### Thermal behaviour

**Figure 6:**
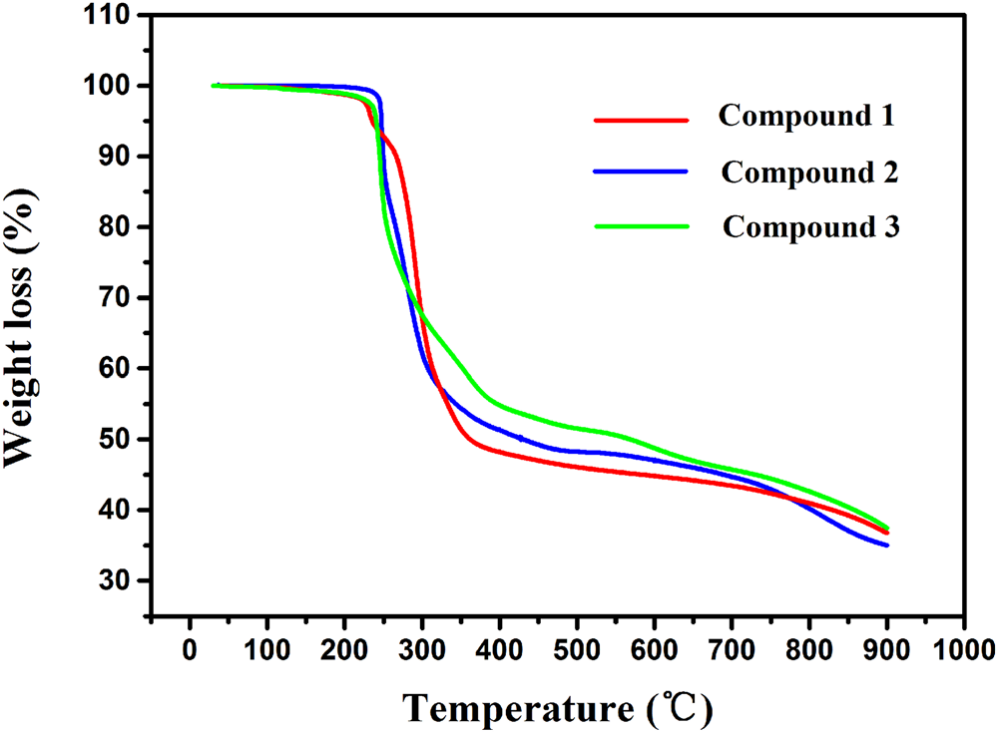
TG curves of compounds **1**–**3**.

The TG behaviours of compounds **1**–**3** were also investigated to examine the thermal stabilities of these stepwise triol-functionalized hybrids, as well as to characterize the compositions of the compounds. Figure 6 presents the temperature (°C) vs. weight-loss (%) curves. Their thermal stabilities were slightly different, owing to the different triol ligands. Compounds **1** and **3** possessed similar thermal stability, displaying instability and decomposition starting at 100 °C, whereas compound **2** showed comparatively good thermal stability, with a thermolysis temperature reaching up to 235 °C. These results indicated that the type of triol ligand may have a considerable effect on the stability of the triol-functionalized hybrids. For compound **1**, the first step at 100–242 °C was assigned to the loss of solvents (Calcd: 6.12%; Found: ca. 6.15%), thus confirming that the solvents were incorporated in the structure, in accordance with the X-ray diffraction results. The second step of ca. 45.62% weight loss corresponded to the expulsion of two TBA counterions and one organic ligand (Calcd: 46.26%). The third step at 400–900 °C resulted from the complete decomposition of the organic triol moiety and the decomposition of the cluster into V_2_O_5_ (residual content: Calcd 36.9%; Found ca. 36.5%). Compound **3** underwent two steps of weight loss. The first step, a loss of 48.88% (Calcd: 49.18%), was ascribed to the loss of two TBA counterions and one organic ligand. In the temperature range of 510–900 °C, compound **3** underwent the second step of weight loss. In this step, bpe was lost first. Then, the organic triol moiety completely decomposed, and finally, the cluster decomposed into V_2_O_5_, together with the partial sublimation of V_2_O_5_ (residual content: Calcd 39.3%; Found ca. 37.5%). Compound **2** underwent a quite different order of decomposition from that of compounds **1** and **3**. It first lost two TBA countercations and two organic ligands at 235–600 °C (Calcd: 54.2%; Found: ca. 53.1%). The final step was the decomposition of the cluster into V_2_O_5_. The calculated residual content was 36.9%, but the experimental content was 35.0%, owing to the partial sublimation of V_2_O_5_.

## Discussion

With the tosyl ester derivative of POMs, a series of organic-functionalized hexavanadate were synthesized by using functional group transformation reactions. Tosylation of pentaerythritol-derivatized hexavanadate here was previously undescribed despite the steric hindrance and electron-withdrawing properties of the POV. Although tosylation of alcohols followed by nucleophilic substitution is a well-established process in organic chemistry, owing to the strong electron-withdrawing properties of POMs, tosylated derivatives of POMs are less likely than pure organic substrates to undergo nucleophilic substitutions. Therefore, excess organic substrates, longer reaction times and higher temperatures are required, and the yields were comparable to that of common post-functionalized hexavanadate hybrid products. What’s more, the nucleophilic substitution of compound **1** with N_3_^−^ and Br^−^ groups verified the feasibility of the synthetic methodology applied for POVs. Finally, to simplify the organic functionalization process of POMs, other organic functionalization approaches will be explored in our laboratories.

## Methods

### Materials

All the chemicals except acetonitrile were used without any further treatment. Acetonitrile was dried by refluxing in the presence of calcium hydride and was distilled before use. The precursor (Bu_4_N)_2_[V_6_O_13_{(OCH_2_)_3_CCH_2_OH}_2_] was synthesized and characterized according to a previously published procedure^48^.

### X-ray crystallography

Single-crystal X-ray diffraction data collection was performed at 173 K, 106 K and 273 K for compound **1**, compound **2** and compound **3**, respectively, by using graphite monochromated Cu K_α_ radiation (*λ* = 1.5418 Å) for compound **1** and compound **3** and Mo K_α_ radiation (*λ* = 0.7107 Å) for compound **2**. Data reduction, cell refinement and experimental absorption correction were performed with Rigaku RAPID AUTO software (Rigaku, 1998, Ver 2.30). The structures were solved by direct methods and refined against *F* ^2^ by the full-matrix least-squares method. All non-hydrogen atoms were refined anisotropically. Hydrogen atoms were generated geometrically. All calculations were carried out in SHELXTL, Ver 5.1^49^, and Olex2, Ver 1.2.8^50^. For compound **2**, the unit cell contained 8 acetonitrile molecules, which were treated as a diffuse contribution to the overall scattering without specific atom positions with SUQEEZE/PLATON. The CCDC numbers of compounds **1**, **2** and **3** are 1520556, 1520557 and 1557515, respectively.

### Mass spectrometry

ESI-MS spectra were recorded with a Thermo Q Exactive spectrometer in acetonitrile solutions (negative mode was used for the experiments, Figures S11–S13).

### NMR spectra

The ^1^H-NMR spectra were recorded using CD_3_CN or DMSO-*d*_6_ as a solvent with a JEOL JNM-EXC 600 spectrometer. The ^13^C-NMR spectra were recorded using DMSO-*d*_6_ as a solvent with a JEOL JNM-EXC 400 spectrometer. The NMR spectra for all compounds are shown in Figures S5–S10.

### Other characterization methods

Fourier transform infrared (FTIR) spectra were obtained using KBr pellets with a PerkinElmer FT-IR spectrometer. The intensities were designated as vs = very strong, s = strong, m = medium, and w = weak (Figures S2–S4). The UV/Vis absorption spectra were recorded on a UN-2100s spectrometer at 298 K (Figure S14). Thermogravimetric analysis (TGA) measurements were investigated with a PerkinElmer TGA-7 instrument with a heating rate of 10 K/min in flowing N_2_ (50.0 mL/min). Elemental analysis was measured with an Elementar Vario EL III element analyser.

### Synthesis of (Bu_4_N)_2_[V_6_O_13_{(OCH_2_)_3_CCH_2_SO_3_C_7_H_4_}_2_]·2.5CH_3_CN, compound 1

(Bu_4_N)_2_[V_6_O_13_{(OCH_2_)_3_CCH_2_OH}_2_] (1.26 g, 1 mmol), TsCl (0.42 g, 2.2 mmol) and DMAP (0.24 g, 2 mmol) were dissolved in 25 mL of freshly distilled acetonitrile with stirring. After Et_3_N (400 µL, 2.85 mmol) was added, the mixture was subsequently stirred for 2 days at 50 °C. The reaction was monitored by ESI-MS. After the reaction was completed, the mixture was cooled to room temperature and filtered into cold water (70 mL) to remove the excess TsCl and ammonium salt formed. The pure products were obtained by recrystallization from a mixture of acetonitrile, DMF and H_2_O with proper volumetric ratios. Suitable orange-yellow block single crystals for X-ray diffraction were grown by slow diffusion of diethyl ether into their acetonitrile solution. The yield was 60% based on V. Elemental analysis for C_61_H_109.5_N_4.5_O_25_S_2_V_6_ (compound **1**): Anal. Calcd: C 42.75, H 6.54, N 1.78, Found: C 42.25, H 6.43, N 2.31. IR (cm^−1^): 2918 m, 2101 m, 1481 m, 1380 w, 1121 w, 1057 m, 952 vs, 807 m, 718 s. ^1^H NMR (400 MHz, CD_3_CN, standardized by solvent peak): δ = 0.95 (24 H, t, J = 7.2 Hz, TBA–H), 1.33 (16H, sextet, TBA–H), 1.58 (16H, quintet, TBA–H), 3.06 (16H, t, J = 8.4 Hz, TBA–H), 2.42 (6H, s, –CH_3_), 3.83 (s, 4H, SO_3_–CH_2_–C), 4.87 (s, 12H, O–CH_2_–C), 7.42 (4H, d, J = 7.8 Hz, CH_phenyl_), 7.74 (4H, d, J = 7.8 Hz, CH_phenyl_). ^13^C NMR (400 MHz, DMSO–d_6_, standardized by solvent peak): δ = 145.74, 132.13, 130.79, 128.27, 82.41, 70.89, 58.09, 23.66, 21.67, 17.74, 14.05. ESI-MS: m/z (%): 1330.00 (6.44%) {(Bu_4_N)[V_6_O_13_{(OCH_2_)_3_CCH_2_SO_3_C_7_H_4_}_2_]}^−^, 543.86 (92.5%) [V_6_O_13_{(OCH_2_)_3_CCH_2_SO_3_C_7_H_4_}_2_]^2−^.

### Synthesis of (Bu_4_N)_2_[V_6_O_13_{(OCH_2_)_3_CCH_2_N_3_}_2_]·4CH_3_CN, compound 2

Compound **2** was prepared by heating a mixture of compound **1** (0.314 g, 0.2 mmol) and NaN_3_ (0.104 g, 1.6 mmol) in 10 mL of dry DMF at 80 °C for 2 days. The reaction was monitored by IR spectroscopy and ESI-MS. After the reaction was completed, the mixture solution was cooled to room temperature, and then, the solvent was completely evaporated. A minimal amount of CH_3_CN was added to the remaining solid to dissolve all the POM materials, and subsequently, the suspension was centrifuged. The precipitates containing excess NaN_3_ and the formed NaOTs were discarded. Suitable orange-red block single crystals for X-ray diffraction were grown by slow diffusion of diethyl ether into their acetonitrile solution. Compound **2** was also easily crystallized in the mother liquor conveniently by addition of a small amount of acetonitrile and water. The yield was 68% based on V. Elemental analysis for C_50_H_100_N_12_O_19_V_6_ (compound **2**): Anal. Calcd: C 40.60, H 6.82, N 11.36, Found: C 39.88, H 6.63, N 10.95. IR (cm^−1^): 3361 w, 2921 m, 2851 m, 1659 w, 1633 m, 1470 m, 1363 w, 1176 m, 1130 w, 1065 m, 952 vs, 804 s, 790 s, 717 s. ^1^H NMR (400 MHz, DMSO–d_6_, standardized by solvent peak): δ = 0.90 (24H, t, J = 7.2 Hz, TBA–H), 1.28 (16H, sextet, TBA–H), 1.53 (16H, quintet, TBA–H), 3.13 (16H, t, J = 8.4 Hz, TBA–H), 3.69 (s, 4H, N_3_–CH_2_–C), 4.83 (s, 12H, O–CH_2_–C). ^13^C NMR (400 MHz, DMSO–d_6_, standardized by solvent peak): δ = 83.62, 58.14, 52.43, 49.57, 23.73, 19.76, 14.06. ESI-MS: m/z (%): 1330.00 (10.71%) {(Bu_4_N)[V_6_O_13_{(OCH_2_)_3_CCH_2_N_3_}_2_]}^−^, 414.85 (98.89%) [V_6_O_13_{(OCH_2_)_3_CCH_2_N_3_}_2_]^2−^.

### Synthesis of (Bu_4_N)_2_[V_6_O_13_{(OCH_2_)_3_CCH_2_Br}_2_], compound 3

Compound **3** was prepared by heating a mixture of compound **1** (0.157 g, 0.1 mmol) and KBr (1.6 mmol, 0.19 g) in 10 mL of dry DMF at 90 °C for 2 days. The reaction was monitored by IR spectroscopy and ESI-MS. After the reaction was completed, the mixture solution was cooled to room temperature, and then, the solvent was completely evaporated. A minimal amount of CH_3_CN was added to the remaining solid to dissolve all the POM materials, and subsequently, the suspension was centrifuged. The precipitates containing excess KBr and the formed KOTs were discarded. Suitable orange-red block single crystals for X-ray diffraction were grown by slow diffusion of diethyl ether into their acetonitrile solution, and the yield was ca. 65% based on V. Elemental analysis for C_42_H_88_Br_2_N_2_O_19_V_6_ (compound **3**): Anal. Calcd: C 36.28, H 6.38, N 2.01, Found: C 35.89, H 6.19, N 1.90. IR (cm^−1^): 2924 m, 1648 w, 1464 m, 1385 w, 1250 w, 1175 w, 1112 s, 1058 s, 947 vs, 802 s, 715 s. ^1^H NMR (600 MHz, CD_3_CN, standardized by solvent peak): δ = 0.95 (24 H, t, J = 7.2 Hz, TBA–H), 1.33 (16H, sextet, TBA–H), 1.58 (16H, quintet, TBA–H), 3.06 (16H, t, J = 8.4 Hz, TBA–H), 2.42 (6H, s, –CH_3_), 3.83 (s, 4H, Br–CH_2_–C), 4.87 (s, 12H, O–CH_2_–C). ^13^C NMR (400 MHz, DMSO–d_6_, standardized by solvent peak): δ = 84.30, 58.08, 37.78, 34.90, 23.63, 19.75, 14.05. ESI-MS: m/z (%): 1147.81 (12.76%) {(Bu_4_N)[V_6_O_13_{(OCH_2_)_3_CCH_2_Br}_2_]}^−^, 452.76 (98.5%) [V_6_O_13_{(OCH_2_)_3_CCH_2_Br}_2_]^2−^.

## Electronic supplementary material

Dataset 3
