# Supplementary material for: Tosylation of alcohols: an effective strategy for the functional group transformation of organic derivatives of polyoxometalates
Source: Sci Rep. 2017 Oct 2;7:12523. doi: 10.1038/s41598-017-12633-8 (PMC5624914; doi:10.1038/s41598-017-12633-8)

checkCIF/PLATON report

You have not supplied any structure factors. As a result the full set of tests cannot be run.

THIS REPORT IS FOR GUIDANCE ONLY. IF USED AS PART OF A REVIEW PROCEDURE FOR PUBLICATION, IT SHOULD NOT REPLACE THE EXPERTISE OF AN EXPERIENCED CRYSTALLOGRAPHIC REFEREE.

No syntax errors found. CIF dictionary Interpreting this report

Datablock: half-sr

Bond precision: C-C = 0.0060 A Wavelength=1.54178

Cell:	a=29.7672(12)	b=16.4119(4)	c=16.6684(5)
	alpha=90	beta=108.645(3)	gamma=90
Temperature:	173 K		

	Calculated	Reported
Volume	7715.8(5)	7715.7(4)
Space group	C 2/c	C 1 2/c 1
Hall group	-C 2yc	-C 2yc
Moiety formula	C24 H30 O25 S2 V6, 2(C16 H36 N)	C24 H30 O25 S2 V6, 2(C16 H36 N)
Sum formula	C56 H102 N2 O25 S2 V6	C61 H109.50 N4.50 O25 S2 V6
Mr	1573.16	1675.79
Dx,g cm-3	1.354	1.443
Z	4	4
Mu (mm-1)	6.946	6.992
F000	3288.0	3508.0
F000’	3301.68	
h,k,lmax	37,20,21	37,20,20
Nref	8105	7965
Tmin,Tmax	0.233,0.497	0.504,1.000
Tmin’	0.098	

Correction method= # Reported T Limits: Tmin=0.504 Tmax=1.000
AbsCorr = MULTI-SCAN

Data completeness= 0.983 Theta(max)= 76.320

R(reflections)= 0.0509(6917) wR2(reflections)= 0.1510(7965)

S = 1.048 Npar= 441

```
The following ALERTS were generated. Each ALERT has the format
       test-name_ALERT_alert-type_alert-level.
Click on the hyperlinks for more details of the test.
```

Alert level B

```
CHEMW03_ALERT_2_B WARNING: The ratio of given/expected molecular weight as
            calculated from the _atom_site* data lies outside
            the range 0.95 <> 1.05
           From the CIF: _cell_formula_units_Z                    4
           From the CIF: _chemical_formula_weight          1675.79
           TEST: Calculate formula weight from _atom_site_*
           atom     mass    num     sum
           C        12.01   56.00  672.62
           H         1.01  102.00  102.82
           N        14.01    2.00   28.01
           O        16.00   25.00  399.97
           S        32.07    2.00   64.13
           V        50.94    6.00  305.65
           Calculated formula weight             1573.20
PLAT043_ALERT_1_B Calculated and Reported Mol. Weight Differ by ..     102.63 Check
PLAT601_ALERT_2_B Structure Contains Solvent Accessible VOIDS of .        149 Ang3
```

Alert level C

```
PLAT041_ALERT_1_C Calc. and Reported SumFormula    Strings  Differ     Please Check
PLAT068_ALERT_1_C Reported F000 Differs from Calcd (or Missing)...     Please Check
PLAT234_ALERT_4_C Large Hirshfeld Difference C27    --  C28A    ..       0.16 Ang.
PLAT241_ALERT_2_C High   'MainMol' Ueq as Compared to Neighbors of        C26 Check
PLAT413_ALERT_2_C Short Inter XH3 .. XHn     H18B   ..  H28E    ..       2.14 Ang.
```

Alert level G

```
FORMU01_ALERT_1_G  There is a discrepancy between the atom counts in the
            _chemical_formula_sum and _chemical_formula_moiety. This is
            usually due to the moiety formula being in the wrong format.
            Atom count from _chemical_formula_sum:   C61 H109.5 N4.5 O25 S2 V6
            Atom count from _chemical_formula_moiety:C56 H102 N2 O25 S2 V6
FORMU01_ALERT_2_G  There is a discrepancy between the atom counts in the
            _chemical_formula_sum and the formula from the _atom_site* data.
            Atom count from _chemical_formula_sum:C61 H109.5 N4.5 O25 S2 V6
            Atom count from the _atom_site data:  C56 H102. N2 O25 S2 V6
CELLZ01_ALERT_1_G Difference between formula and atom_site contents detected.
CELLZ01_ALERT_1_G ALERT: Large difference may be due to a
            symmetry error - see SYMMG tests
           From the CIF: _cell_formula_units_Z    4
           From the CIF: _chemical_formula_sum  C61 H109.50 N4.50 O25 S2 V6
           TEST: Compare cell contents of formula and atom_site data

           atom    Z*formula  cif sites diff
           C        244.00    224.00   20.00
           H        438.00    408.00   30.00
           N         18.00      8.00   10.00
           O        100.00    100.00    0.00
           S          8.00      8.00    0.00
           V         24.00     24.00    0.00
PLAT128_ALERT_4_G Alternate Setting for Input Space Group     C2/c       I2/a Note
PLAT301_ALERT_3_G Main Residue  Disorder ............ Percentage =          3 Note
PLAT380_ALERT_4_G Incorrectly? Oriented X(sp2)-Methyl Moiety .....         C1 Check
PLAT710_ALERT_4_G Delete 1-2-3 or 2-3-4 Linear Torsion Angle ... #          2 Do !
            V2  -V3  -O12 -V3    -30.00  3.00   7.556   1.555   1.555   7.556
```

```
PLAT710_ALERT_4_G Delete 1-2-3 or 2-3-4 Linear Torsion Angle ... #        13 Do !
            V2  -V1  -O12 -V1   -112.00  4.00   7.556   1.555   1.555   7.556
PLAT710_ALERT_4_G Delete 1-2-3 or 2-3-4 Linear Torsion Angle ... #        20 Do !
            V3  -V2  -O12 -V2     31.00 15.00   7.556   1.555   1.555   7.556
PLAT710_ALERT_4_G Delete 1-2-3 or 2-3-4 Linear Torsion Angle ... #        32 Do !
            V3  -V1  -O12 -V1    -17.00  4.00   7.556   1.555   1.555   7.556
PLAT710_ALERT_4_G Delete 1-2-3 or 2-3-4 Linear Torsion Angle ... #        39 Do !
            V1  -V2  -O12 -V2    126.00 15.00   7.556   1.555   1.555   7.556
PLAT710_ALERT_4_G Delete 1-2-3 or 2-3-4 Linear Torsion Angle ... #        50 Do !
            V1  -V3  -O12 -V3   -125.00  3.00   7.556   1.555   1.555   7.556
PLAT710_ALERT_4_G Delete 1-2-3 or 2-3-4 Linear Torsion Angle ... #        76 Do !
            O8  -V2  -O12 -V2    -59.00 15.00   1.555   1.555   1.555   7.556
PLAT710_ALERT_4_G Delete 1-2-3 or 2-3-4 Linear Torsion Angle ... #        89 Do !
            O8  -V1  -O12 -V1     73.00  4.00   1.555   1.555   1.555   7.556
PLAT710_ALERT_4_G Delete 1-2-3 or 2-3-4 Linear Torsion Angle ... #        95 Do !
            O7  -V2  -O12 -V2   -144.00 15.00   1.555   1.555   1.555   7.556
PLAT710_ALERT_4_G Delete 1-2-3 or 2-3-4 Linear Torsion Angle ... #       106 Do !
            O7  -V3  -O12 -V3    145.00  3.00   1.555   1.555   1.555   7.556
PLAT710_ALERT_4_G Delete 1-2-3 or 2-3-4 Linear Torsion Angle ... #       114 Do !
            O5  -V3  -O12 -V3     60.00  3.00   1.555   1.555   1.555   7.556
PLAT710_ALERT_4_G Delete 1-2-3 or 2-3-4 Linear Torsion Angle ... #       124 Do !
            O5  -V1  -O12 -V1    158.00  4.00   1.555   1.555   1.555   7.556
PLAT710_ALERT_4_G Delete 1-2-3 or 2-3-4 Linear Torsion Angle ... #       129 Do !
            O13 -V3  -O12 -V3   -128.00  3.00   1.555   1.555   1.555   7.556
PLAT710_ALERT_4_G Delete 1-2-3 or 2-3-4 Linear Torsion Angle ... #       140 Do !
            O13 -V1  -O12 -V1    -20.00  4.00   7.556   1.555   1.555   7.556
PLAT710_ALERT_4_G Delete 1-2-3 or 2-3-4 Linear Torsion Angle ... #       145 Do !
            O11 -V2  -O12 -V2    124.00 15.00   1.555   1.555   1.555   7.556
PLAT710_ALERT_4_G Delete 1-2-3 or 2-3-4 Linear Torsion Angle ... #       159 Do !
            O11 -V1  -O12 -V1   -115.00  4.00   7.556   1.555   1.555   7.556
PLAT710_ALERT_4_G Delete 1-2-3 or 2-3-4 Linear Torsion Angle ... #       164 Do !
            O4  -V1  -O12 -V2   -116.90  0.50   1.555   1.555   1.555   7.556
PLAT710_ALERT_4_G Delete 1-2-3 or 2-3-4 Linear Torsion Angle ... #       165 Do !
            O4  -V1  -O12 -V2     63.10  0.50   1.555   1.555   1.555   1.555
PLAT710_ALERT_4_G Delete 1-2-3 or 2-3-4 Linear Torsion Angle ... #       166 Do !
            O4  -V1  -O12 -V3    -32.20  0.50   1.555   1.555   1.555   1.555
PLAT710_ALERT_4_G Delete 1-2-3 or 2-3-4 Linear Torsion Angle ... #       167 Do !
            O4  -V1  -O12 -V3    147.80  0.50   1.555   1.555   1.555   7.556
PLAT710_ALERT_4_G Delete 1-2-3 or 2-3-4 Linear Torsion Angle ... #       168 Do !
            O4  -V1  -O12 -V1    131.00  4.00   1.555   1.555   1.555   7.556
PLAT710_ALERT_4_G Delete 1-2-3 or 2-3-4 Linear Torsion Angle ... #       173 Do !
            O9  -V2  -O12 -V2    -87.00 15.00   1.555   1.555   1.555   7.556
PLAT710_ALERT_4_G Delete 1-2-3 or 2-3-4 Linear Torsion Angle ... #       174 Do !
            O9  -V2  -O12 -V3     62.10  0.50   1.555   1.555   1.555   1.555
PLAT710_ALERT_4_G Delete 1-2-3 or 2-3-4 Linear Torsion Angle ... #       175 Do !
            O9  -V2  -O12 -V3   -117.90  0.50   1.555   1.555   1.555   7.556
PLAT710_ALERT_4_G Delete 1-2-3 or 2-3-4 Linear Torsion Angle ... #       176 Do !
            O9  -V2  -O12 -V1    -33.40  0.50   1.555   1.555   1.555   1.555
PLAT710_ALERT_4_G Delete 1-2-3 or 2-3-4 Linear Torsion Angle ... #       177 Do !
            O9  -V2  -O12 -V1    146.60  0.50   1.555   1.555   1.555   7.556
PLAT710_ALERT_4_G Delete 1-2-3 or 2-3-4 Linear Torsion Angle ... #       184 Do !
            O10 -V2  -O12 -V2     28.00 15.00   1.555   1.555   1.555   7.556
PLAT710_ALERT_4_G Delete 1-2-3 or 2-3-4 Linear Torsion Angle ... #       196 Do !
            O10 -V3  -O12 -V3    -32.00  3.00   7.556   1.555   1.555   7.556
PLAT710_ALERT_4_G Delete 1-2-3 or 2-3-4 Linear Torsion Angle ... #       204 Do !
            O6  -V3  -O12 -V2    144.60  0.60   1.555   1.555   1.555   7.556
PLAT710_ALERT_4_G Delete 1-2-3 or 2-3-4 Linear Torsion Angle ... #       205 Do !
            O6  -V3  -O12 -V2    -35.40  0.60   1.555   1.555   1.555   1.555
PLAT710_ALERT_4_G Delete 1-2-3 or 2-3-4 Linear Torsion Angle ... #       206 Do !
            O6  -V3  -O12 -V3    115.00  3.00   1.555   1.555   1.555   7.556
PLAT710_ALERT_4_G Delete 1-2-3 or 2-3-4 Linear Torsion Angle ... #       207 Do !
            O6  -V3  -O12 -V1   -120.00  0.60   1.555   1.555   1.555   7.556
```

```
PLAT710_ALERT_4_G Delete 1-2-3 or 2-3-4 Linear Torsion Angle ... #        208 Do !
            O6  -V3  -O12 -V1     60.00  0.60   1.555   1.555   1.555   1.555
PLAT764_ALERT_4_G Overcomplete CIF Bond List Detected (Rep/Expd) .       1.21 Ratio
PLAT779_ALERT_4_G Suspect or Irrelevant (Bond) Angle in CIF .... #        279 Check
              C24A -C23  -H23F    1.555   1.555   1.555             11.70 Deg.
PLAT779_ALERT_4_G Suspect or Irrelevant (Bond) Angle in CIF .... #        280 Check
              C24A -C23  -H23C    1.555   1.555   1.555             42.00 Deg.
PLAT779_ALERT_4_G Suspect or Irrelevant (Bond) Angle in CIF .... #        295 Check
              H23B -C23  -H23E    1.555   1.555   1.555              6.00 Deg.
PLAT779_ALERT_4_G Suspect or Irrelevant (Bond) Angle in CIF .... #        298 Check
              H23B -C23  -H23D    1.555   1.555   1.555             42.20 Deg.
PLAT779_ALERT_4_G Suspect or Irrelevant (Bond) Angle in CIF .... #        302 Check
              H23F -C23  -H23C    1.555   1.555   1.555             37.00 Deg.
PLAT779_ALERT_4_G Suspect or Irrelevant (Bond) Angle in CIF .... #        307 Check
              C24C -C23  -H23A    1.555   1.555   1.555             23.90 Deg.
PLAT860_ALERT_3_G Number of Least-Squares Restraints ............          1 Note
```

---

   0 **ALERT level A** = Most likely a serious problem - resolve or explain
   3 **ALERT level B** = A potentially serious problem, consider carefully
   5 **ALERT level C** = Check. Ensure it is not caused by an omission or oversight
  48 **ALERT level G** = General information/check it is not something unexpected

   6 ALERT type 1 CIF construction/syntax error, inconsistent or missing data
   5 ALERT type 2 Indicator that the structure model may be wrong or deficient
   2 ALERT type 3 Indicator that the structure quality may be low
  43 ALERT type 4 Improvement, methodology, query or suggestion
   0 ALERT type 5 Informative message, check

It is advisable to attempt to resolve as many as possible of the alerts in all categories. Often the minor alerts point to easily fixed oversights, errors and omissions in your CIF or refinement strategy, so attention to these fine details can be worthwhile. In order to resolve some of the more serious problems it may be necessary to carry out additional measurements or structure refinements. However, the purpose of your study may justify the reported deviations and the more serious of these should normally be commented upon in the discussion or experimental section of a paper or in the "special_details" fields of the CIF. checkCIF was carefully designed to identify outliers and unusual parameters, but every test has its limitations and alerts that are not important in a particular case may appear. Conversely, the absence of alerts does not guarantee there are no aspects of the results needing attention. It is up to the individual to critically assess their own results and, if necessary, seek expert advice.

Publication of your CIF in IUCr journals

A basic structural check has been run on your CIF. These basic checks will be run on all CIFs submitted for publication in IUCr journals (*Acta Crystallographica*, *Journal of Applied Crystallography*, *Journal of Synchrotron Radiation*); however, if you intend to submit to *Acta Crystallographica Section C* or *E* or *IUCrData*, you should make sure that full publication checks are run on the final version of your CIF prior to submission.

Publication of your CIF in other journals

Please refer to the *Notes for Authors* of the relevant journal for any special instructions relating to CIF submission.

PLATON version of 11/08/2016; check.def file version of 04/08/2016

Datablock half-sr - ellipsoid plot

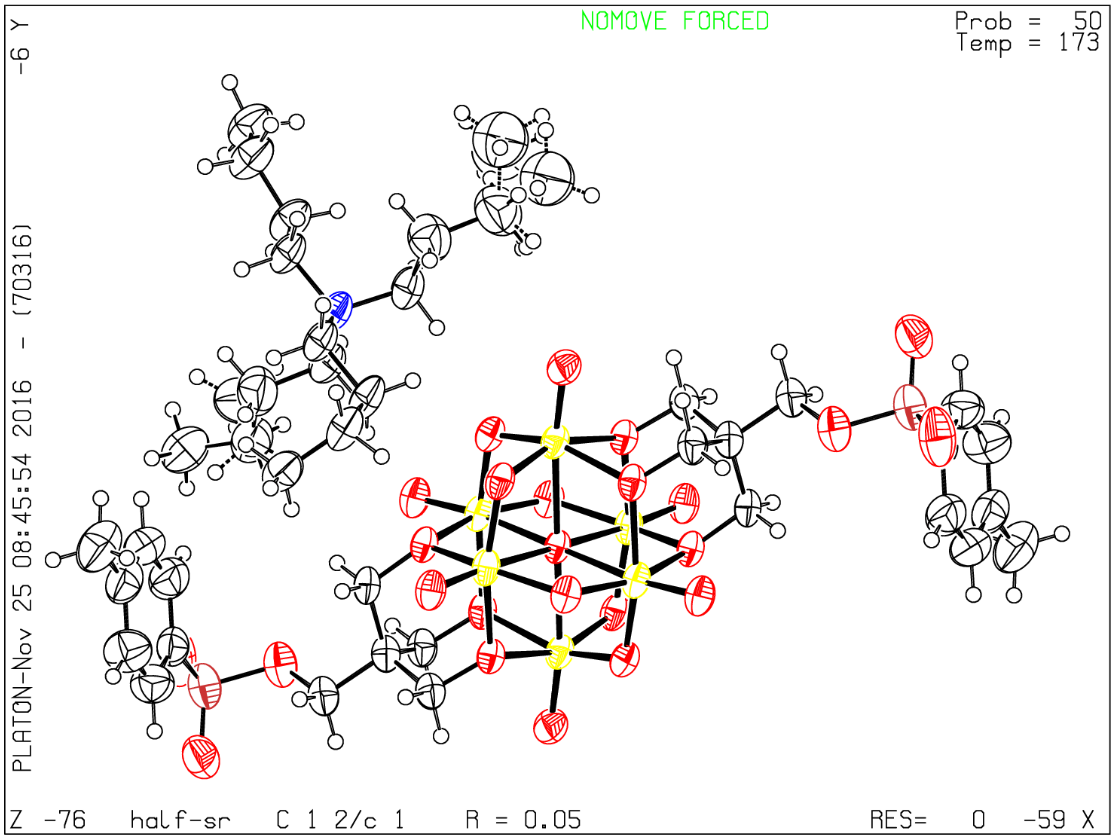

Supplement: Supplementary file 1 — Dataset 3 [file 41598_2017_12633_MOESM1_ESM.zip › cif/Compound 1-checkcif.pdf]
