# Supplementary material for: Tosylation of alcohols: an effective strategy for the functional group transformation of organic derivatives of polyoxometalates
Source: Sci Rep. 2017 Oct 2;7:12523. doi: 10.1038/s41598-017-12633-8 (PMC5624914; doi:10.1038/s41598-017-12633-8)

checkCIF/PLATON report

Structure factors have been supplied for datablock(s) exp_3751_p-sr

THIS REPORT IS FOR GUIDANCE ONLY. IF USED AS PART OF A REVIEW PROCEDURE FOR PUBLICATION, IT SHOULD NOT REPLACE THE EXPERTISE OF AN EXPERIENCED CRYSTALLOGRAPHIC REFEREE.

No syntax errors found. CIF dictionary Interpreting this report

Datablock: exp_3751_p-sr

Bond precision: C-C = 0.0065 A Wavelength=0.71073

Cell:	a=28.0514(15)	b=16.1942(6)	c=16.8286(6)
	alpha=90	beta=115.432(4)	gamma=90
Temperature:	106 K		

	Calculated	Reported
Volume	6903.9(6)	6903.9(6)
Space group	C 2/c	C 1 2/c 1
Hall group	-C 2yc	-C 2yc
Moiety formula	C10 H16 N6 O19 V6, 2(C15.47 H33.34 N), 1.064(C H3), 2.128(H) [+	C10 H16 N6 O19 V6, 2(C16 H36 N)
Sum formula	C42 H88 N8 O19 V6 [+ solvent]	C46 H102 N12 O19 V6
Mr	1314.84	1433.03
Dx,g cm-3	1.265	1.379
Z	4	4
Mu (mm-1)	0.841	0.849
F000	2744.0	3008.0
F000’	2752.32	
h,k,lmax	38,22,23	37,21,23
Nref	9710	8159
Tmin,Tmax	0.707,0.880	0.899,1.000
Tmin’	0.595	

Correction method= # Reported T Limits: Tmin=0.899 Tmax=1.000
AbsCorr = MULTI-SCAN

Data completeness= 0.840 Theta(max)= 29.596

R(reflections)= 0.0632(5952) wR2(reflections)= 0.1783(8159)

S = 1.055 Npar= 358

The following ALERTS were generated. Each ALERT has the format
test-name_ALERT_alert-type_alert-level.
Click on the hyperlinks for more details of the test.

Alert level B

```
CHEMW03_ALERT_2_B WARNING: The ratio of given/expected molecular weight as
            calculated from the _atom_site* data lies outside
            the range 0.95 <> 1.05
           From the CIF: _cell_formula_units_Z                    4
           From the CIF: _chemical_formula_weight          1433.03
           TEST: Calculate formula weight from _atom_site_*
           atom     mass    num     sum
           C        12.01   42.00  504.46
           H         1.01   88.00   88.70
           N        14.01    8.00  112.06
           O        16.00   19.00  303.98
           V        50.94    6.00  305.65
           Calculated formula weight             1314.85
PLAT910_ALERT_3_B Missing # of FCF Reflection(s) Below Theta(Min)          12 Note
PLAT990_ALERT_1_B Deprecated RES file style based SQUEEZE job ....          ! Note
```

Alert level C

```
PLAT223_ALERT_4_C Solv./Anion Resd 2   H   Ueq(max)/Ueq(min) Range        4.4 Ratio
PLAT245_ALERT_2_C U(iso) H28A    Smaller than U(eq) C28     by ...      0.024 AngSq
PLAT250_ALERT_2_C Large U3/U1 Ratio for Average U(i,j) Tensor ....        2.7 Note
PLAT341_ALERT_3_C Low Bond Precision on  C-C Bonds ...............    0.00646 Ang.
PLAT906_ALERT_3_C Large K value in the Analysis of Variance ......      6.131 Check
PLAT911_ALERT_3_C Missing # FCF Refl Between THmin & STh/L=  0.600          3 Report
PLAT918_ALERT_3_C Reflection(s) with I(obs) much Smaller I(calc) .          1 Check
PLAT934_ALERT_3_C Number of (Iobs-Icalc)/SigmaW > 10 Outliers ....          1 Check
```

Alert level G

```
FORMU01_ALERT_1_G  There is a discrepancy between the atom counts in the
            _chemical_formula_sum and _chemical_formula_moiety. This is
            usually due to the moiety formula being in the wrong format.
            Atom count from _chemical_formula_sum:   C46 H102 N12 O19 V6
            Atom count from _chemical_formula_moiety:C42 H88 N8 O19 V6
FORMU01_ALERT_2_G  There is a discrepancy between the atom counts in the
            _chemical_formula_sum and the formula from the _atom_site* data.
            Atom count from _chemical_formula_sum:C46 H102 N12 O19 V6
            Atom count from the _atom_site data:  C42 H88.00495 N8 O19 V6
CELLZ01_ALERT_1_G Difference between formula and atom_site contents detected.
CELLZ01_ALERT_1_G ALERT: Large difference may be due to a
            symmetry error - see SYMMG tests
           From the CIF: _cell_formula_units_Z    4
           From the CIF: _chemical_formula_sum  C46 H102 N12 O19 V6
           TEST: Compare cell contents of formula and atom_site data

           atom    Z*formula  cif sites diff
           C        184.00    168.00   16.00
           H        408.00    352.00   56.00
           N         48.00     32.00   16.00
           O         76.00     76.00    0.00
           V         24.00     24.00    0.00
PLAT002_ALERT_2_G Number of Distance or Angle Restraints on AtSite         10 Note
PLAT012_ALERT_1_G No     _shelx_res_checksum found in CIF ........     Please Check
PLAT041_ALERT_1_G Calc. and Reported SumFormula    Strings  Differ     Please Check
```

```
PLAT042_ALERT_1_G Calc. and Reported MoietyFormula Strings  Differ     Please Check
PLAT068_ALERT_1_G Reported F000 Differs from Calcd (or Missing)...     Please Check
PLAT083_ALERT_2_G SHELXL Second Parameter in WGHT  Unusually Large       7.68 Why ?
PLAT128_ALERT_4_G Alternate Setting for Input Space Group     C2/c       I2/a Note
PLAT164_ALERT_4_G Nr. of Refined C-H H-Atoms in Heavy-Atom Struct.          2 Note
PLAT171_ALERT_4_G The CIF-Embedded .res File Contains EADP Records          1 Report
PLAT172_ALERT_4_G The CIF-Embedded .res File Contains DFIX Records          1 Report
PLAT176_ALERT_4_G The CIF-Embedded .res File Contains SADI Records          2 Report
PLAT300_ALERT_4_G Atom Site Occupancy of <N7     is Constrained at       0.35 Check
PLAT300_ALERT_4_G Atom Site Occupancy of <N8     is Constrained at       0.35 Check
PLAT300_ALERT_4_G Atom Site Occupancy of <N9     is Constrained at       0.35 Check
PLAT300_ALERT_4_G Atom Site Occupancy of <N10    is Constrained at       0.35 Check
PLAT300_ALERT_4_G Atom Site Occupancy of <N11    is Constrained at        0.3 Check
PLAT300_ALERT_4_G Atom Site Occupancy of <N12    is Constrained at        0.3 Check
PLAT301_ALERT_3_G Main Residue  Disorder ..............(Resd  1)..         10 % Note
PLAT302_ALERT_4_G Anion/Solvent/Minor-Residue Disorder (Resd  2)..         15 % Note
PLAT302_ALERT_4_G Anion/Solvent/Minor-Residue Disorder (Resd  3)..        100 % Note
PLAT302_ALERT_4_G Anion/Solvent/Minor-Residue Disorder (Resd  4)..        100 % Note
PLAT304_ALERT_4_G Non-Integer Number of Atoms (  49.81) in Resd. #          2 Check
PLAT304_ALERT_4_G Non-Integer Number of Atoms (   0.95) in Resd. #          3 Check
PLAT304_ALERT_4_G Non-Integer Number of Atoms (   1.18) in Resd. #          4 Check
PLAT304_ALERT_4_G Non-Integer Number of Atoms (   0.29) in Resd. #          5 Check
PLAT304_ALERT_4_G Non-Integer Number of Atoms (   0.29) in Resd. #          6 Check
PLAT304_ALERT_4_G Non-Integer Number of Atoms (   0.24) in Resd. #          7 Check
PLAT304_ALERT_4_G Non-Integer Number of Atoms (   0.24) in Resd. #          8 Check
PLAT432_ALERT_2_G Short Inter X...Y Contact  C28    ..  C3B     ..       3.20 Ang.
PLAT606_ALERT_4_G VERY LARGE Solvent Accessible VOID(S) in Structure        ! Info
PLAT720_ALERT_4_G Number of Unusual/Non-Standard Labels ..........         20 Note
PLAT764_ALERT_4_G Overcomplete CIF Bond List Detected (Rep/Expd) .       1.24 Ratio
PLAT779_ALERT_4_G Suspect or Irrelevant (Bond) Angle in CIF .... #        212 Check
              C0AA -C28  -C0AB    1.555   1.555   1.555            20.50 Deg.
PLAT802_ALERT_4_G CIF Input Record(s) with more than 80 Characters          2 Info
PLAT860_ALERT_3_G Number of Least-Squares Restraints .............          9 Note
PLAT869_ALERT_4_G ALERTS Related to the use of SQUEEZE Suppressed           ! Info
PLAT912_ALERT_4_G Missing # of FCF Reflections Above STh/L=  0.600       1472 Note
PLAT913_ALERT_3_G Missing # of Very Strong Reflections in FCF ....          3 Note
PLAT955_ALERT_1_G Reported (CIF) and Actual (FCF) Lmax Differ by .          1 Units
PLAT961_ALERT_5_G Dataset Contains no Negative Intensities .......     Please Check
PLAT978_ALERT_2_G Number C-C Bonds with Positive Residual Density.          1 Note
```

```
   0 ALERT level A = Most likely a serious problem - resolve or explain
   3 ALERT level B = A potentially serious problem, consider carefully
   8 ALERT level C = Check. Ensure it is not caused by an omission or oversight
  45 ALERT level G = General information/check it is not something unexpected

   9 ALERT type 1 CIF construction/syntax error, inconsistent or missing data
   8 ALERT type 2 Indicator that the structure model may be wrong or deficient
   9 ALERT type 3 Indicator that the structure quality may be low
  29 ALERT type 4 Improvement, methodology, query or suggestion
   1 ALERT type 5 Informative message, check
```

It is advisable to attempt to resolve as many as possible of the alerts in all categories. Often the minor alerts point to easily fixed oversights, errors and omissions in your CIF or refinement strategy, so attention to these fine details can be worthwhile. In order to resolve some of the more serious problems it may be necessary to carry out additional measurements or structure refinements. However, the purpose of your study may justify the reported deviations and the more serious of these should normally be commented upon in the discussion or experimental section of a paper or in the "special_details" fields of the CIF. checkCIF was carefully designed to identify outliers and unusual parameters, but every test has its limitations and alerts that are not important in a particular case may appear. Conversely, the absence of alerts does not guarantee there are no aspects of the results needing attention. It is up to the individual to critically assess their own results and, if necessary, seek expert advice.

Publication of your CIF in IUCr journals

A basic structural check has been run on your CIF. These basic checks will be run on all CIFs submitted for publication in IUCr journals (*Acta Crystallographica*, *Journal of Applied Crystallography*, *Journal of Synchrotron Radiation*); however, if you intend to submit to *Acta Crystallographica Section C* or *E* or *IUCrData*, you should make sure that full publication checks are run on the final version of your CIF prior to submission.

Publication of your CIF in other journals

Please refer to the *Notes for Authors* of the relevant journal for any special instructions relating to CIF submission.

PLATON version of 24/11/2016; check.def file version of 23/11/2016

Datablock exp_3751_p-sr - ellipsoid plot

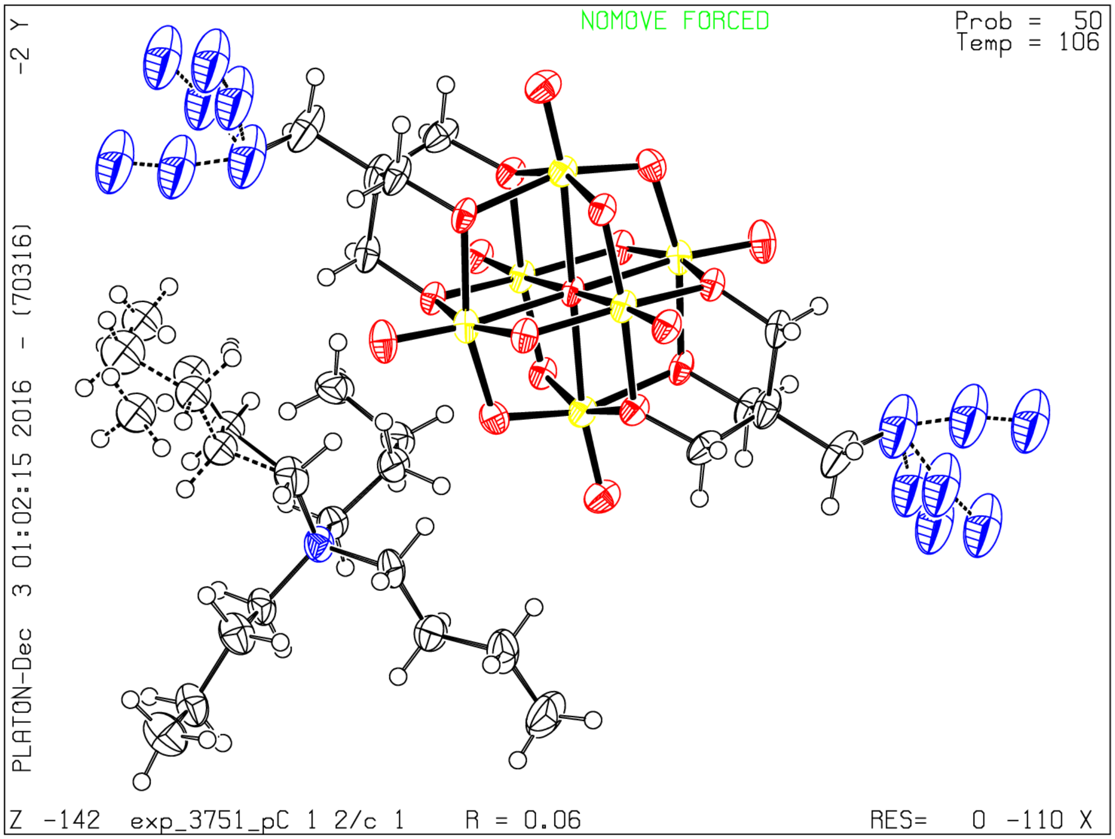

Supplement: Supplementary file 1 — Dataset 3 [file 41598_2017_12633_MOESM1_ESM.zip › cif/Compound 2-checkcif.pdf]
