# Supplementary material for: Tosylation of alcohols: an effective strategy for the functional group transformation of organic derivatives of polyoxometalates
Source: Sci Rep. 2017 Oct 2;7:12523. doi: 10.1038/s41598-017-12633-8 (PMC5624914; doi:10.1038/s41598-017-12633-8)

checkCIF/PLATON report

You have not supplied any structure factors. As a result the full set of tests cannot be run.

THIS REPORT IS FOR GUIDANCE ONLY. IF USED AS PART OF A REVIEW PROCEDURE FOR PUBLICATION, IT SHOULD NOT REPLACE THE EXPERTISE OF AN EXPERIENCED CRYSTALLOGRAPHIC REFEREE.

No syntax errors found.   CIF dictionary   Interpreting this report

Datablock: exp_1369

Bond precision: C-C = 0.0109 A   Wavelength=1.54184

Cell:	a=10.8872(2)	b=13.0882(4)	c=20.4111(6)
	alpha=81.547(3)	beta=83.464(2)	gamma=85.946(2)
Temperature:	298 K		

	Calculated	Reported
Volume	2853.85(13)	2853.86(14)
Space group	P -1	P -1
Hall group	-P 1	-P 1
Moiety formula	C10 H16 Br2 O19 V6, 2(C16 H36 N)	2(C5 H8 Br O9.5 V3), 2(C16 H36 N)
Sum formula	C42 H88 Br2 N2 O19 V6	C42 H88 Br2 N2 O19 V6
Mr	1390.58	1390.60
Dx,g cm-3	1.618	1.618
Z	2	2
Mu (mm-1)	10.131	10.131
F000	1428.0	1428.0
F000’	1429.80	
h,k,lmax	13,16,25	13,16,25
Nref	11927	11512
Tmin,Tmax	0.132,0.363	0.577,1.000
Tmin’	0.032	

Correction method= # Reported T Limits: Tmin=0.577 Tmax=1.000
AbsCorr = MULTI-SCAN

Data completeness= 0.965   Theta(max)= 75.950

R(reflections)= 0.0685(9120)   wR2(reflections)= 0.2153(11512)

S = 1.052   Npar= 660

The following ALERTS were generated. Each ALERT has the format
test-name_ALERT_alert-type_alert-level.
Click on the hyperlinks for more details of the test.

Alert level B

```
PLAT230_ALERT_2_B Hirshfeld Test Diff for    Br1    --  C5      ..        7.3 s.u.
```

Alert level C

```
PLAT234_ALERT_4_C Large Hirshfeld Difference C33    --  C34     ..       0.22 Ang.
PLAT242_ALERT_2_C Low    'MainMol' Ueq as Compared to Neighbors of        C10 Check
PLAT341_ALERT_3_C Low Bond Precision on  C-C Bonds ...............    0.01088 Ang.
PLAT360_ALERT_2_C Short  C(sp3)-C(sp3) Bond   C27    -   C28    ..       1.37 Ang.
PLAT360_ALERT_2_C Short  C(sp3)-C(sp3) Bond   C31    -   C32    ..       1.35 Ang.
PLAT360_ALERT_2_C Short  C(sp3)-C(sp3) Bond   C33    -   C34    ..       1.42 Ang.
PLAT361_ALERT_2_C Long   C(sp3)-C(sp3) Bond   C32    -   C33    ..       1.66 Ang.
```

Alert level G

```
PLAT005_ALERT_5_G No Embedded Refinement Details found  in the CIF     Please Do !
PLAT042_ALERT_1_G Calc. and Reported MoietyFormula Strings  Differ     Please Check
PLAT072_ALERT_2_G SHELXL First  Parameter in WGHT  Unusually Large       0.12 Report
PLAT300_ALERT_4_G Atom Site Occupancy of Br2A    is Constrained at        0.8 Check
PLAT300_ALERT_4_G Atom Site Occupancy of Br2B    is Constrained at        0.2 Check
PLAT300_ALERT_4_G Atom Site Occupancy of H10C    is Constrained at       0.75 Check
PLAT300_ALERT_4_G Atom Site Occupancy of H10D    is Constrained at       0.75 Check
PLAT300_ALERT_4_G Atom Site Occupancy of H10A    is Constrained at       0.25 Check
PLAT300_ALERT_4_G Atom Site Occupancy of H10B    is Constrained at       0.25 Check
PLAT301_ALERT_3_G Main Residue  Disorder ..............(Resd  1)..          5 % Note
PLAT764_ALERT_4_G Overcomplete CIF Bond List Detected (Rep/Expd) .       1.24 Ratio
```

---

0 **ALERT level A** = Most likely a serious problem - resolve or explain
1 **ALERT level B** = A potentially serious problem, consider carefully
7 **ALERT level C** = Check. Ensure it is not caused by an omission or oversight
11 **ALERT level G** = General information/check it is not something unexpected

1 ALERT type 1 CIF construction/syntax error, inconsistent or missing data
7 ALERT type 2 Indicator that the structure model may be wrong or deficient
2 ALERT type 3 Indicator that the structure quality may be low
8 ALERT type 4 Improvement, methodology, query or suggestion
1 ALERT type 5 Informative message, check

It is advisable to attempt to resolve as many as possible of the alerts in all categories. Often the minor alerts point to easily fixed oversights, errors and omissions in your CIF or refinement strategy, so attention to these fine details can be worthwhile. In order to resolve some of the more serious problems it may be necessary to carry out additional measurements or structure refinements. However, the purpose of your study may justify the reported deviations and the more serious of these should normally be commented upon in the discussion or experimental section of a paper or in the "special_details" fields of the CIF. checkCIF was carefully designed to identify outliers and unusual parameters, but every test has its limitations and alerts that are not important in a particular case may appear. Conversely, the absence of alerts does not guarantee there are no aspects of the results needing attention. It is up to the individual to critically assess their own results and, if necessary, seek expert advice.

Publication of your CIF in IUCr journals

A basic structural check has been run on your CIF. These basic checks will be run on all CIFs submitted for publication in IUCr journals (*Acta Crystallographica*, *Journal of Applied Crystallography*, *Journal of Synchrotron Radiation*); however, if you intend to submit to *Acta Crystallographica Section C* or *E* or *IUCrData*, you should make sure that full publication checks are run on the final version of your CIF prior to submission.

Publication of your CIF in other journals

Please refer to the *Notes for Authors* of the relevant journal for any special instructions relating to CIF submission.

PLATON version of 27/03/2017; check.def file version of 24/03/2017

Datablock exp_1369 - ellipsoid plot

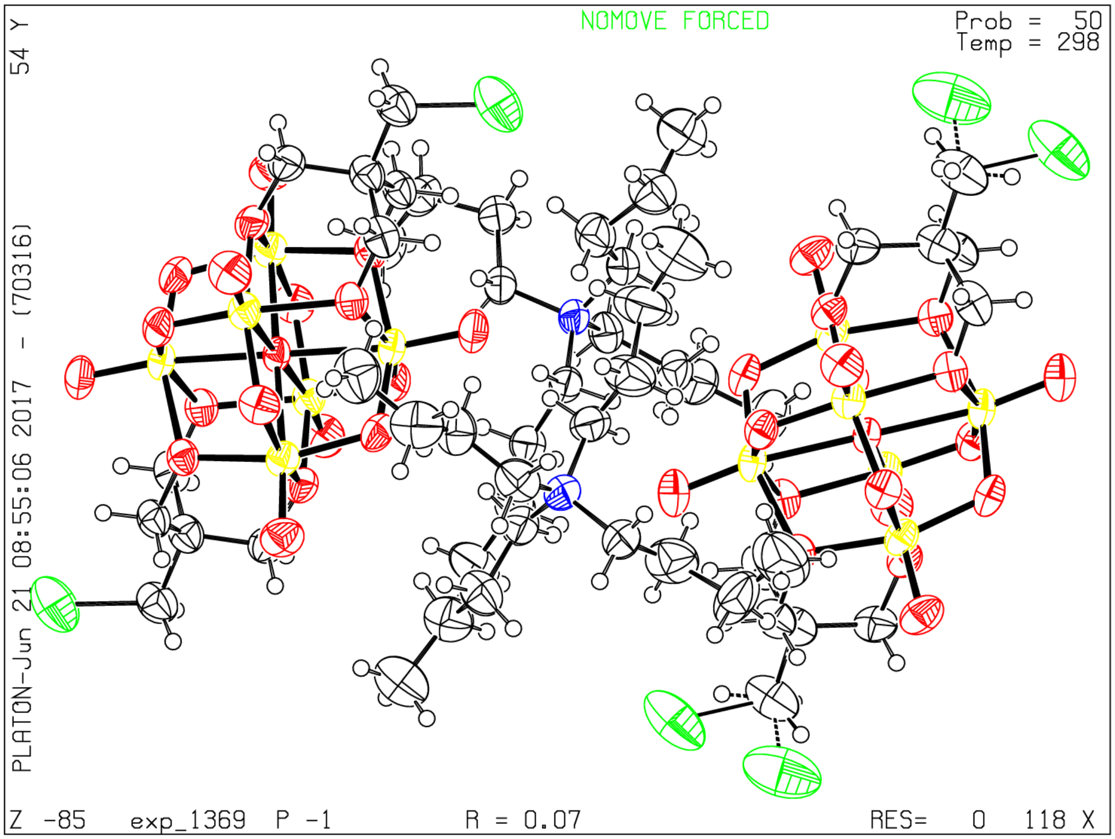

Supplement: Supplementary file 1 — Dataset 3 [file 41598_2017_12633_MOESM1_ESM.zip › cif/Compound 3-checkcif.pdf]
